# Performance of broiler chicken fed multicarbohydrases supplemented low energy diet

**DOI:** 10.14202/vetworld.2017.727-731

**Published:** 2017-07-02

**Authors:** Kumar Govil, Sunil Nayak, R. P. S. Baghel, A. K. Patil, C. D. Malapure, Dinesh Thakur

**Affiliations:** Department of Animal Nutrition, College of Veterinary Science and Animal Husbandry, Jabalpur - 482 001, Madhya Pradesh, India

**Keywords:** broiler, carcass traits, multicarbohydrases, performance

## Abstract

**Aim:**

Objective of this study was to investigate the effect of multicarbohydrases supplementation on performance of broilers fed low energy diet.

**Materials and Methods:**

A total of 75 days old chicks were selected and randomly divided into three treatments groups (T_1_, T_2_, and T_3_); each group contained 25 chicks distributed in five replicates of five chicks each. T_1_ group (positive control) was offered control ration formulated as per Bureau of Indian Standards recommendations. In T_2_ group (negative control) ration, metabolizable energy (ME) was reduced by 100 kcal/kg diet. T_3_ group ration was same as that of T_2_ except that it was supplemented with multicarbohydrases (xylanase at 50 g/ton+mannanase at 50 g/ton+amylase at 40 g/ton). Feed intake and body weight of all experimental birds were recorded weekly. Metabolic trial was conducted for 3 days at the end of experiment to know the retention of nutrients.

**Results:**

Significant improvement (p<0.01) was observed in total weight gain, feed conversion efficiency, and performance index in broilers under supplementary group T_3_ as compared to T_1_ and T_2_ groups. Retention of crude protein and ether extract was significantly increased (p<0.05) in T_3_ group supplemented with multicarbohydrases as compared to other groups. Retention of dry matter, crude fiber, and nitrogen-free extract was comparable in all the three groups. Significantly highest dressed weight, eviscerated weight, and drawn weight (% of live body weight) were observed in multicarbohydrases supplemented T_3_ group, however it was comparable in T_1_ and T_2_ groups.

**Conclusion:**

It was concluded that the supplementation of multicarbohydrases (xylanase at 50 g/ton+mannanase at 50 g/ton+amylase at 40 g/ton) in low energy diet improved overall performance of broilers.

## Introduction

Corn is a highly digestible source of energy and it also provides various other nutrients including amino acids. It is main source of energy in poultry diets on a global scale including India and its inclusion rate in commercial diets can be up to 70%. Corn and soybean meal are two major ingredients in commercial poultry diets in many parts of the world which contain lower concentration of antinutritive high molecular weight-soluble nonstarch polysaccharides (NSP) that can impede normal digestion and absorption processes of nutrients including carbohydrates and proteins in the digestive tract. A combination of undigested fat, protein, and starch contributes to energy loss and use of exogenous enzymes can be a good strategy to make this energy available to birds. About 400-450 kcal of energy per kg of diet is not digested when birds are fed a typical corn-soya ration [1]. Nowadays, various exogenous enzymes are being used in poultry diets to improve feed utilization. The exogenous enzymes are used either to correct the lack of specific endogenous enzymes for digesting certain nutrients in various feedstuffs or to hydrolyze antinutritional factors in feed ingredients.

The use of exogenous feed enzymes in poultry diets is becoming popular to overcome the adverse effects of antinutritional factors and to improve digestion of dietary components and bird performance [2]. The NSP degrading enzyme products can enhance the access of endogenous enzymes to nutrients (e.g., starch granules) by releasing the nutrients from complex cell wall molecules [3]. Supplemental multicarbohydrases (amylase, xylanase, and mannase) increased the digestibility of nutrients in broiler chicken fed corn-based diets [4-6].

The existing knowledge on the role of exogenous enzyme in enhancing the feeding value of corn-soy diets in poultry is not only limited but also inconsistent. Keeping in view this brief background, this study was planned to investigate the effect of multicarbohydrases (exogenous enzyme) supplementation on performance of broilers fed low energy diet.

## Materials and Methods

### Ethical approval

The experimental design and plan of this study strictly followed the norms of the Institutional Animal Ethics Committee of Nanaji Deshmukh Veterinary Sciences University Jabalpur, Madhya Pradesh.

### Study area

The proposed experiment was conducted in the Department of Animal Nutrition, College of Veterinary Science and Animal Husbandry, Jabalpur, Madhya Pradesh.

### Experimental design

A total of 75 days old cobb 400 chicks were randomly divided into three treatments groups (T_1_, T_2_ and T_3_); each group contained 25 chicks distributed in five replicates of five chicks each. T_1_ group (positive control) was offered control corn-soy based ration in mash form formulated as per Bureau of Indian Standards [7] recommendations. In T_2_ group (negative control) ration, metabolizable energy (ME) was reduced by 100 kcal/kg diet. T_3_ group ration was same as that of T_2_ except that it was supplemented with commercially available multicarbohydrases (xylanase at 50 g/ton+mannanase at 50 g/ton+amylase at 40 g/ton). All the three enzymes, i.e., xylanase (1,200,000 IU/g), mannase (200,000 IU/g), and amylase (160,000 IU/g) were purchased individually and incorporated in the feed. Enzymes were heat stable and able to with stand pelletization temperature up to 90°C for 90 s. The doses of enzymes were decided on the basis of review of literature available.

Ingredients and nutrient composition of all the experimental diets for prestarter, starter and finisher phase are presented in Table-1. Total experimental period was of 6 weeks.

**Table-1:** Ingredients and nutrient composition of different broiler diets.

Attributes	Prestarter diet			Starter diet			Finisher diet		
		
	T_1_	T_2_	T_3_	T_1_	T_2_	T_3_	T_1_	T_2_	T_3_
Ingredient composition (%)									
Maize	49.58	49.58	54.78	50.13	49.58	49.58	54.78	54.78	54.78
Soya bean cake	41.50	41.50	34.50	39.80	41.50	41.50	34.50	34.50	34.50
Oil	4.50	4.39	6.60	6.00	5.89	5.89	6.71	6.60	6.60
LSP	1.30	1.30	1.20	1.25	1.30	1.30	1.20	1.20	1.20
DCP	1.75	1.75	1.70	1.75	1.75	1.75	1.70	1.70	1.70
Methionine	0.30	0.30	1.00	0.30	0.30	0.30	1.00	1.00	1.00
Lysine	0.23	0.23	0.60	0.23	0.23	0.23	0.60	0.60	0.60
Salt	0.35	0.35	0.35	0.35	0.35	0.35	0.35	0.35	0.35
Trace mineral premix*	0.12	0.12	0.12	0.12	0.12	0.12	0.12	0.12	0.12
Vitamin premix**	0.03	0.03	0.03	0.03	0.03	0.03	0.03	0.03	0.03
Nutrient composition analyzed (%)									
CP	23.20	23.16	23.10	22.20	22.26	22.18	20.26	20.20	20.10
Ca	1.09	1.02	1.08	1.07	1.09	1.06	1.02	1.07	1.04
Total P	0.77	0.72	0.75	0.75	0.77	0.75	0.77	0.75	0.76
Nutrient composition calculated									
Energy (kcal ME/kg diet)	3000	2900	2900	3100	3000	3000	3200	3100	3100
Lysine %	1.33	1.33	1.33	1.24	1.24	1.24	1.18	1.18	1.18
Methionine %	0.52	0.52	0.52	0.54	0.54	0.54	0.47	0.47	0.47

* Each kg of trace mineral premix contains: Cu - 15 g; Co - 02 g; Fe - 60 g; Zn - 80 g; Mn - 80 g; I - 02 g; Se - 0.3 g.

** Each kg of vitamin premix contains: Vitamin A - 80 MIU; vitamin D_3_ - 12 MIU; vitamin E - 70 g; vitamin K_3_ - 8 g; vitamin B_1_ - 6.4 g; vitamin B_2_ - 40 g; vitamin B_6_ - 12.8 g; vitamin B_12_ - 160 mg; Nicotinic acid - 80 g; Folic acid - 4 g; Biotin - 24 mg. CP=Crude protein, LSP=Limestone powder, DCP=Dicalcium phosphate

The experimental chicks were reared in the battery brooder house. The battery brooders were cleaned washed and fumigated using formaldehyde and potassium permanganate four days before start of the experiment. Artificial heat was provided to chicks during the early period of growth using electric bulbs. In addition, room heaters were also used to maintain the room temperature as the experiment was conducted in spring season. Mash feed was offered *ad libitum* to the broilers in feeders. Care was taken that feeders are full of feed at all time and constant watch was kept to avoid feed wastage. An ample supply of clean and fresh drinking water was made available to the birds all the time through simple water channel type waterer.

### Data recording

All the experimental chicks were weighed at the beginning of the experiment and subsequently at weekly intervals to estimate total weight gain, average daily gain, and feed efficiency ratio (FER). Weekly feed consumption of broilers was recorded replicate wise on the basis of feed offered and left over at the end of that week. Performance index (PI) was calculated as per the formula given below proposed by Bird [8].

PI=Body weight gain (g)×FER

The metabolic trial was conducted for 3 days at the end of experiment to know the retention of nutrients (dry matter [DM], crude protein [CP], ether extract [EE], crude fiber [CF], and nitrogen free extract [NFE]). To study the carcass traits (dressed weight, eviscerated weight, and drawn weight), two broilers in each replicate were slaughtered on termination of the experiment.

### Analytical procedures

Nutrient compositions of feed and fecal sample were analyzed as per methods described in Association of Official Analytical Chemists [9].

### Statistical analysis

The data obtained during experiment were analyzed statistically using the methods described by Snedecor and Cochran [10]. Differences among the treatments were tested for significance by Duncan’s new multiple range test.

## Results

### Performance of broilers

The observations regarding weight gain (g/bird), feed intake (g/bird), FER, and performance index of broilers in all the three treatment groups (T_1_, T_2_, and T_3_) are presented in Table-2. Supplementation of multicarbohydrases enzyme in the diets of broilers influenced their body weight gain significantly. Minimum and significantly (p<0.05) lower body weight gain (2240.60±87.97) was observed in broilers assigned negative control (T_2_) diet. However, when the broilers fed energy deficient diet supplemented with multicarbohydrases (T_3_), the body weight gain (2663.20±59.31) was improved significantly (p<0.05). Feed intake (g/bird) was statistically similar in all the three groups irrespective of dietary treatments.

**Table-2:** Performance of broilers as influenced by multicarbohydrases supplementation (0-6 weeks).

Attributes	Treatments		

	T_1_	T_2_	T_3_
Weight gain (g/bird)	2517.40^b^±18.50	2240.60^c^±87.97	2663.20^a^±59.31
Feed intake (g/bird)	4780±25.30	4825±19.75	4640±30.25
FER	0.527^b^±0.01	0.464^c^±0.02	0.574^a^±0.01
Performance index	1326.00^b^±22.62	1041.16^c^±10.91	1530.23^a^±52.41

a,b,c Mean±standard error bearing similar superscripts in the same row does not differ significantly (p<0.05). FER=Feed efficiency ratio

Supplementation of multicarbohydrases enzyme in the diets of broilers significantly influenced their feed efficiency. Minimum and significantly (p<0.05) lower FER (0.464±0.02) was observed in broilers assigned negative control (T_2_) diet. When the broilers fed low energy diet was supplemented with multicarbohydrases (T_3_), the FER (0.574±0.01) was improved significantly (p<0.05). Minimum and significantly (p<0.05) lower performance index (1041.16±10.91) was observed in broilers assigned negative control (T_2_) diet, however it was improved significantly (p<0.05) in T_3_ group (1530.23±52.41) supplemented with multicarbohydrases.

### Retention of nutrients (%)

The observations regarding retention of nutrients (%) in various groups are presented in Table-3. Minimum and significantly (p<0.05) lower CP (57.73±1.19) and EE (55.02±1.19) retention (%) was observed in broilers of T_2_ group assigned negative control diet. However, maximum and significantly (p<0.05) higher CP (64.45±1.01) and EE (63.65±1.32) retention (%) was observed in broilers of T_3_ group supplemented with multicarbohydrases. Retention of other nutrients, i.e., DM, CF, and NFE was not affected by the supplementation of multicarbohydrases in broiler diet.

**Table-3:** Retention of nutrients (%) and carcass yields (% of live weight) in broilers as influenced by multicarbohydrases supplementation (0-6 weeks).

Attributes	Treatments		

	T_1_	T_2_	T_3_
Retention of nutrients (%)			
DM	68.39±2.46	66.50±0.97	70.04±0.87
CP	61.39^ab^±1.15	57.73^b^±1.19	64.45^a^±1.01
EE	61.53^b^±1.33	55.02^c^±1.19	63.65^a^±1.32
CF	52.51±0.76	49.80±0.55	54.50±1.25
NFE	84.60±1.50	78.45±0.80	89.75±1.25
Carcass yields (% of live weight)			
Dressed weight	79.59^b^±1.4	76.31^b^±1.22	84.41^a^±1.29
Eviscerated weight	69.58^b^±3.35	66.44^b^±1.65	72.05^a^±1.43
Drawn weight	72.25^b^±1.61	70.59^b^±1.39	79.62^a^±1.29

a,b,c Mean±standard error bearing similar superscripts in the same row does not differ significantly (p<0.05). DM=Dry matter, EE=Ether extract, CP=Crude protein, CF=Crude fiber, NFE=Nitrogen free extract

### Carcass traits

The observations regarding carcass yield (% of live weight) in various groups are presented in Table-3. Maximum and significantly (p<0.05) highest dressed, eviscerated and drawn weights (% of live weight) were recorded in T_3_ groups broilers fed multicarbohydrases supplemented energy deficit diet, however it remained statistically similar in T_1_ and T_2_ groups.

## Discussion

Minimum and significantly lowest weight gain (g/bird) was recorded in group T_2_ in which ME was reduced by 100 kcal/kg diet however; weight gain was improved significantly after multicarbohydrases supplementation in T_3_ group fed energy deficit diet. Addition of multicarbohydrases to maize-based diet increases ileal and total tract digestibilities of CP and starch which could the direct reason for the improvement of growth performance in broilers [11]. Results regarding significantly higher weight gain in multicarbohydrases supplemented group are consistent with Cowieson and Adeola [12] who reported 14% improvement in weight gain after supplementing enzyme cocktail (xylanase, amylase, protease, and phytase) in broilers fed nutritionally marginal diets. Jose *et al*. [13] also reported improvement in body weight gain of broilers after supplementation of carbohydrases (amylase+xylanase) in negative control diet. Similar results were also reported by Avila *et al*. [14] and Zeng *et al*. [15] where they stated that the supplementation with both NSP-degrading enzymes (xylanase and β-glucanase) and phytase to the broiler diets increased their body weight.

The results of this study indicated that when the broilers were fed low energy density diet supplemented with carbohydrases enzymes (xylanase 50 g/ton+mannanase 50 g/ton+amylase 40 g/ton), feed intake (g/bird) was not influenced statistically. In support this, Zhang *et al*. [11] also reported that supplementation of multicarbohydrases enzyme did not affect the feed intake of broilers but increase their body weight gain significantly indicating improved feed efficiency.

In this study, significantly better (p<0.05) feed efficiency and performance index were observed in broilers assigned low energy diet supplemented with multicarbohydrases (T_3_); however, minimum and significantly lower (p<0.05) feed efficiency and performance index were observed in T_2_ group fed negative control diet. Similar results were reported by Zanella *et al*. [16], who observed improved FCR in broiler chickens fed multicarbohydrases enzyme supplemented corn-soy diet. These results regarding better feed efficiency and performance index in multicarbohydrases supplemented group are also supported by Jose *et al*. [13].

Retention (%) of CP and EE was significantly (p<0.05) improved in broilers fed diets supplemented with multicarbohydrases (xylanase 50 g/ton+mannanase 50 g/ton+amylase 40 g/ton). These results are in agreements with Olukosi *et al*. [17] who reported that the supplementation of enzyme cocktail in the negative control diet improved total tract retention of nutrients. Tang *et al*. [18] reported that supplementation of combination of enzymes (xylanase, amylase, and protease) significantly (p<0.05) improved apparent ileal digestibility of CP, which is in agreement with our results. Similarly, Zhang *et al*. [11] also reported that addition of enzyme (xylanase) to maize-based diet has the capacity to increase ileal and total tract digestibilities of CP and starch which could be the direct reason for the improvement of growth performance in broilers. Retention of other nutrients, *viz*., DM, CF, and NFE was not influenced by supplementation of multicarbohydrases in this study.

In present study significantly (p<0.05) higher carcass yield (% of live weight) in terms of dressed weight, eviscerated weight and drawn weight was found in broilers fed low energy diet supplemented with multicarbohydrases (xylanase 50 g/ton+mannanase 50 g/ton+amylase 40 g/ton). In support to present results, Khan *et al*. [19] reported significantly improved (p<0.05) dressing percentage in broilers fed carbohydrases treated sunflower-corn based diets compared to unsupplemented one. Yuan *et al*. [20] also stated that enzyme supplementation to broilers diet improved their dressing percentage. Similarly, Wang *et al*. [21] and Hajati *et al*. [22] reported increased carcass yield in broilers by addition of enzymes in diet attributable to higher fat deposition in carcass. Recently Mishra *et al*. [23] and Imran *et al*. [24] also reported maximum value of dressing percentage in broilers fed carbohydrases supplemented diet.

## Conclusion

From the results of the present experiment, it was concluded that multicarbohydrases (xylanase at 50 g/ton+mannanase at 50 g/ton+amylase at 40 g/ton) supplementation improved overall performance of broilers fed low energy diets.

## Authors’ Contributions

SN and RPSB have designed the plan of work. KG conducted the experiment, carried out the laboratory work and analyzed the results. AKP and CDM drafted and DT revised the manuscript. All the authors read and approved the final manuscript.
